# Varicella zoster virus infections in neurological patients: a clinical study

**DOI:** 10.1186/s12879-018-3137-2

**Published:** 2018-05-25

**Authors:** Thomas Skripuletz, Kaweh Pars, Alina Schulte, Philipp Schwenkenbecher, Özlem Yildiz, Tina Ganzenmueller, Maike Kuhn, Annette Spreer, Ulrich Wurster, Refik Pul, Martin Stangel, Kurt-Wolfram Sühs, Corinna Trebst

**Affiliations:** 1 Department of Neurology, Hannover Medical School, Carl-Neuberg-Str-1, 30625 Hannover, Germany; 2 Institute of Virology, Hannover Medical School, Hannover, Germany; 3 TWINCORE Centre for Experimental and Clinical Infection Research, Hannover and Helmholtz Centre for Infection Research, Braunschweig, Germany; 4 Department of Neurology, University Medical Center of Mainz, Mainz, Germany; 5 Department of Neurology, University Clinic Essen, Essen, Germany

**Keywords:** Herpes zoster, VZV, Cerebrospinal fluid, CNS

## Abstract

**Background:**

Varicella zoster virus (VZV) reactivation is a common infectious disease in neurology and VZV the second most frequent virus detected in encephalitis. This study investigated characteristics of clinical and laboratory features in patients with VZV infection.

**Methods:**

Two hundred eighty two patients with VZV reactivation that were hospitalized in the department of neurology in the time from 2005 to 2013 were retrospectively evaluated. Results from cerebrospinal fluid (CSF) analysis were available from 85 patients.

**Results:**

Trigeminal rash was the most common clinical manifestation, followed by segmental rash, CNS infection, facial nerve palsy, postherpetic neuralgia, and radiculitis. MRI of the brain performed in 25/33 patients with encephalitis/meningitis did not show any signs of infection in the brain parenchyma. Only one patient showed contrast enhancement in the hypoglossal nerve. General signs of infection such as fever or elevated CRP values were found in only half of the patients. Furthermore, rash was absent in a quarter of patients with CNS infection and facial nerve palsy, and thus, infection could only be proven by CSF analysis. Although slight inflammatory CSF changes occurred in few patients with isolated rash, the frequency was clearly higher in patients with CNS infection and facial nerve palsy.

**Conclusion:**

Monosegmental herpes zoster is often uncomplicated and a diagnostic lumbar puncture is not essential. In contrast, CSF analysis is an essential diagnostic tool in patients with skin lesions and cranial nerve or CNS affection. In patients with neuro-psychiatric symptoms and inflammatory CSF changes analysis for VZV should be performed even in the absence of skin lesions.

## Background

Varicella zoster virus (VZV) reactivation is one of the most common neurological infectious diseases and VZV the second most frequent virus causing encephalitis or meningitis [1–3]. It develops by reactivation of latently persistent virus in the nerve ganglia after primary infection with chickenpox. Since the immunological control of the virus is mainly T-cell mediated, reactivation often occurs with aging or due to immunosuppression [4]. The incidence varies from 2 to 4.6 per 1000 person-years to 10 per 1000 person-years in patients above the age of 80 [5]. Since the virus is highly restricted to humans, animal models are challenging [6, 7], and thus, our knowledge mainly derives from the clinical setting. The clinical presentation varies from mild self-limiting monosegmental cutaneous affection to severe encephalitis with a mortality of up to 12–15% [2, 8]. The mortality risk further increases in immunocompromised patients being especially high in patients following allogeneic hematopoietic stem cell transplantation who frequently develop a VZV dissemination (17%) resulting in a mortality of up to 50% [9]. Further complications include vasculopathy, panuveitis, and postherpetic neuralgia [10, 11].

The typical segmental rash usually allows a quick clinical diagnosis. However, if rash is absent, diagnosis can be delayed or missed in patients with facial nerve palsy or CNS infection. Furthermore, even in patients with VZV infection of the CNS cranial MRI shows mostly age-appropriate findings (80%) and does not help to verify the diagnosis [12]. Cerebrospinal fluid (CSF) analysis including detection of the virus either by PCR or by measuring virus-specific intrathecal synthesis of IgG antibodies is required in such cases [13, 14].

Most of the available CSF data are based on patients with VZV infection of the CNS where CSF pleocytosis is a common feature [15–17]. In these patients increased concentrations of glial fibrillary acidic protein and the light subunit of neurofilament protein were found, but did not correlate with the outcome [18]. However, CSF abnormalities including pleocytosis (46%) have been reported in patients with VZV reactivation without any CNS symptoms but still correlated with acute complications [19]. Because clinical and CSF findings in patients with VZV infection are still poorly defined, we performed a thorough evaluation of clinical and laboratory data with special emphasis on CSF.

## Methods

### Patients

Data were retrospectively collected from 282 patients that were treated as inpatients for VZV disease at the Department of Neurology of the Hannover Medical School between 2005 and 2013. Data search between 2005 and 2013 was performed for the following terms: varicella, zoster, VZV, chickenpox, rash, infection, encephalitis, meningitis, myelitis, facial palsy, bell’s palsy, postherpetic, neuralgia, radiculitis. 30,136 patient charts were screened and 282 patients were included in the study. Patients were categorized into the following groups: trigeminal nerve ganglionitis with isolated segmental rash (V1, V2, or V3), dorsal root ganglionitis with isolated segmental rash (cervical, thoracic, lumbar, or sacral region) or combined with radiculitis and neuronal affection, facial nerve palsy, CNS infection (encephalitis, meningitis, myelitis), and postherpetic neuralgia. Results from CSF analysis were available from 85 patients (30.1% of all patients): 34/34 patients with CNS infection, 19/25 patients (76%) with facial nerve palsy, 21/65 patients (32.3%) with dorsal root ganglionitis, and 11/142 patients (7.7%) with trigeminal nerve ganglionitis. MRI examinations of the brain were performed by using 1.5 Tesla machines and included T1-weighted, T2-weighted, fluid-attenuation inversion recovery (FLAIR), and diffusion-weighted (DWI) sequences in 23 patients. In two patients T1-weighted, T2-weighted and FLAIR sequences were performed. In one patient T1-weighted and T2-weighted sequences were performed. Contrast-enhanced T1-weighted sequences were performed in 23 patients.

One patient included in the present study was previously described in detail [20]. The investigation was approved by the Institutional Ethics Committee of the Hannover Medical School.

### CSF analytical procedures

CSF and serum were analysed by standard methods [21–23]. CSF-blood barrier function was assessed by calculating age-corrected albumin quotients (QAlb = CSF albumin/serum albumin) [24]. Intrathecal synthesis of IgG, IgA, and IgM was calculated based on the method of Reiber-Felgenhauer referring the IgG, IgA, and IgM quotients to QAlb [24]. CSF-specific oligoclonal bands were determined by isoelectric focusing in polyacrylamide gels with consecutive silver staining. VZV infection of the CSF was diagnosed by either quantitative real-time PCR analysis [25] and/or detection of virus-specific intrathecal synthesis of IgG antibodies (Enzygnost ELISA kit, Siemens Healthcare) and calculation of the VZV-specific antibody index (AI) according to [24] with a cut-off ≥1.5 [26].

### Statistical analysis

We used contingency tables to analyze dichotomous variables in “signs of infection” (Table 1) and “CSF findings” (Table 2) and Fisher’s exact test to assess statistical significance. Contingency tables were used for the groups of concomitant diseases. Statistical significance between two of these groups were determined by Fisher’s exact test. Kruskal-Wallis-Test in combination with Dunn’s post test was used to compare values of different groups in CSF findings. *P*-values < 0.05 were considered statistically significant.

**Table 1:** General signs of inflammation defined as fever (> 38 °C) and elevated CRP (> 8 mg/l) are shown in patients with varicella zoster virus reactivation. The third column shows the amount of patients with normal temperature and normal CRP values

Patients	Temperature > 38 °C	Elevated CRP	Normal temperature and normal CRP
Trigeminal nerve ganglionitis (*n* = 142)	9 (6%)	54 (38%)	86 (60%)
Dorsal root ganglionitis (*n* = 65)	1 (2%)	19 (29%)	46 (71%)
with rash only (*n* = 60)	1 (2%)	17 (28%)	43 (72%)
with radiculitis (*n* = 5)	0	2 (40%)	3 (60%)
Facial nerve palsy (*n* = 25)	0	9 (36%)	16 (64%)
CNS infection (*n* = 34)	10 (29%)	17 (50%)	14 (41%)
Encephalitis (*n* = 18)	8 (44%)	14 (78%)	2 (11%)
Meningitis (*n* = 15)	2 (13%)	2 (13%)	12 (80%)
Postherpetic neuralgia (*n* = 16)	0	6 (38%)	10 (63%)

**Table 2:** Cerebrospinal fluid laboratory findings in all punctured patients diagnosed with varicella zoster virus reactivation

Parameter	Trigeminal nerve ganglionitis with rash (*n* = 11)	Dorsal root ganglionitis with rash (*n* = 16)	Dorsal root ganglionitis with radiculitis (*n* = 5)	Facial nerve palsy (*n* = 19)	CNS infection (*n* = 34)
Pleocytosis (≥5 cells/μL)	2 (18%)	2 (13%)	4 (80%)	12 (63%)	32 (94%)
Lactate (≥3.5 mmol/L)	0	0	0	1 (5%)	3 (9%)
Blood-CSF barrier dysfunction	0	8 (50%)	3 (60%)	8 (42%)	20 (59%)
Intrathecal synthesis of immunoglobulins	0	0	0	4 (21%)	6 (18%)
Isolated IgG	_	_	_	1	_
Isolated IgA	_	_	_	_	_
Isolated IgM	_	_	_	_	_
Combined IgG + IgM	_	_	_	_	_
Combined IgG + IgA	_	_	_	_	1
Combined IgM + IgA	_	_	_	2	1
Combined IgG + IgM + IgA	_	_	_	1	4
CSF oligoclonal bands	1 (9%)	0	1 (20%)	7 (37%)	11 (32%)
PCR/VZV antibody synthesis	0	2 (13%)	3 (60%)	12 (63%)	34 (100%)
PCR positive	_	1	1	3	23
VZV antibody synthesis	_	1	2	9	11

## Results

### Clinical manifestations of VZV disease

#### Trigeminal nerve ganglionitis with isolated rash

**Fig. 1:**
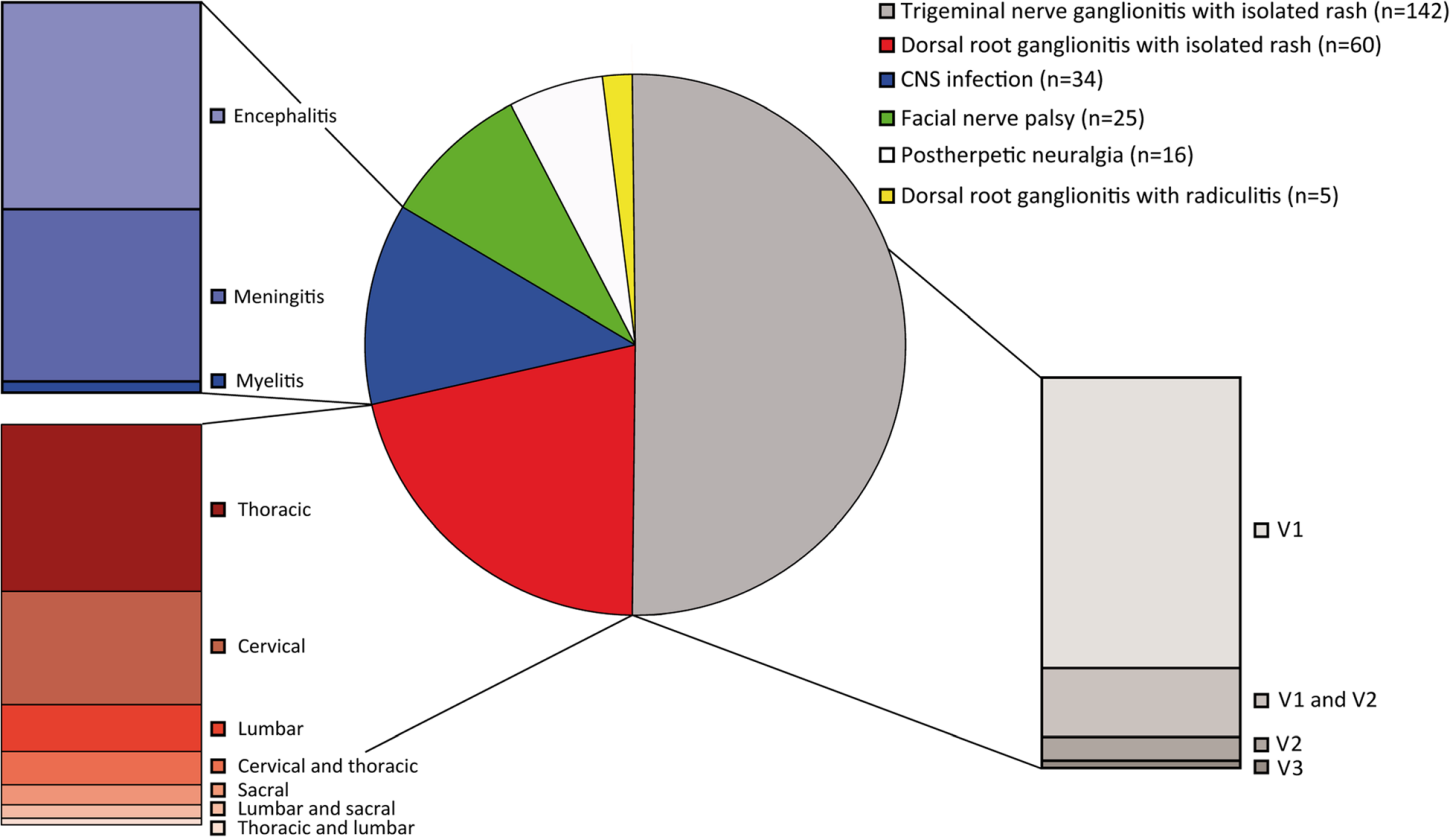
Distribution of 282 patients with varicella zoster virus reactivation. Patients suffered from trigeminal nerve ganglionitis with segmental rash (V1, V1 + V2, V2, or V3), dorsal root ganglionitis with segmental rash (cervical, thoracic, lumbar, sacral region, or a combination of two segments), facial nerve palsy, CNS infection (encephalitis, meningitis, or myelitis), postherpetic neuralgia, and dorsal root ganglionitis with segmental rash and radiculitis with neuronal affection. In the first two groups (trigeminal and dorsal root ganglionitis), patients were included that presented with skin affection only. Patients with skin lesions combined with facial nerve palsy or CNS infection were included in the last two groups in order to avoid double identification

**Fig. 2:**
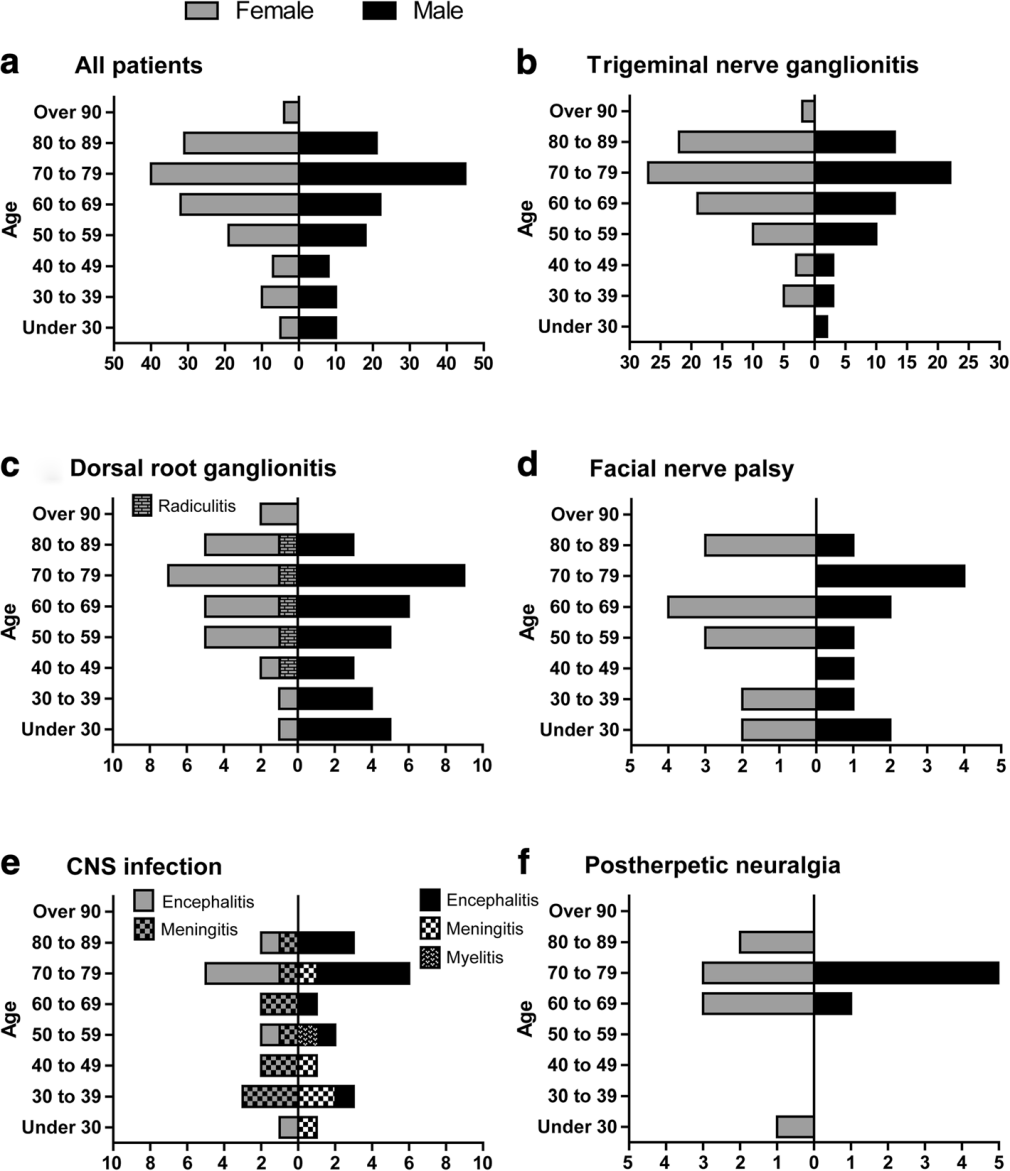
Age and gender distribution of varicella zoster virus reactivation in the study population (**a**-**f**). The age distribution shows that predominantly patients above the age of 50 peaking in the eight decade of life were diseased. There was no gender difference. Patients with trigeminal root ganglionitis and dorsal root ganglionitis were predominantly diseased and showed skin lesions only (**b**, **c**). In five female patients, dorsal root ganglionitis with segmental skin lesions was accompanied by nerve affection due to radiculitis (marked in **c**). In the CNS infection group patients with encephalitis, meningitis, and myelitis were marked separately (**e**). The graph E shows that patients with encephalitis were older as compared to patients with meningitis. Graphs show numbers of female and male patients distributed in life decades

Ganglionitis of the trigeminal nerve was the most common VZV infection in our cohort (50% of all patients, Fig. 1). The gender distribution was 57% females to 43% males (Fig. 2). The ophthalmic branch of the trigeminal nerve (V1) was the most frequently affected one (90%). Most of these patients were infected in the V1 region only (72%) while the combination of affected V1 and V2 branches was found in 19% of patients in this group. The V2 and V3 divisions alone were rarely involved as only 10 patients (7%) presented a VZV infection in the V2 region and three patients (2%) were found with an infected V3 area. The combination of V1 + V3 or V2 + V3 was not seen.

#### Dorsal root ganglionitis

Dorsal root ganglionitis with isolated rash was found in 60 patients (21%). The gender distribution was 42% females to 58% males (Fig. 2). Herpes zoster was most common in the thoracic region (46%), followed by the cervical region (32%), lumbar region (15%), and sacral region (7%). While most of these patients were infected in one region, 8 patients showed zoster rash in two adjacent regions simultaneously: cervical + thoracic region (5 patients), thoracic + lumbar region (1 patient), lumbar + sacral region (2 patients).

In 5 female patients dorsal root ganglionitis with segmental rash was accompanied by nerve affection due to radiculitis (2%, Fig. 2). Four patients with rash in the L5 dermatome showed weakness of foot elevation. Another patient with sacral zoster suffered from an Elsberg syndrome with urinary and bowel dysfunction.

#### Facial nerve palsy

Facial nerve palsy was found in 25 patients (9%). The gender distribution was 52% females to 48% males. Twelve patients (48%) showed rash or reported pain in the affected ear region, while another 6 patients (24%) showed rash in the ophthalmic region of the trigeminal nerve. Seven patients (28%) presented with facial nerve palsy only without any rash or pain.

#### CNS infection (encephalitis, meningitis, myelitis)

Infection of the CNS was found in 34 patients (12%). The gender distribution was 47% females to 53% males. Eighteen patients suffered from encephalitis (53% in this group), 15 patients (44%) from meningitis, and 1 patient (3%) from myelitis.

Rash was found in 26 patients with CNS infection (76%). Rash was found more frequently in patients with encephalitis (16 patients; 89%) than in patients with meningitis (9 patients, 60%). Nineteen patients (56%) presented with trigeminal nerve ganglionitis (18 were infected in the V1 region and 1 in the V3 region), while 7 patients (21%) had a rash corresponding to dorsal root ganglionitis (1 cervical, 2 thoracic, 3 lumbar, and 1 sacral region).

In the encephalitis group one patient presented with a new movement disorder (myoclonus of both arms) without any additional symptoms. All other patients showed disturbance of consciousness and/or neuropsychiatric symptoms (confusion, disorientation, cognitive disorder, behavioural disorder, psychomotor deceleration, hallucination). In addition to neuropsychiatric symptoms, 1 patient showed a hemichorea, 1 patient presented with focal seizures, 2 patients showed oculomotor nerve palsy, and 1 patient showed affection of multiple cranial nerves (VII, IX, X, and XII).

Of the patients with meningitis, all but one suffered from headache only. One patient showed vertigo due to involvement of cranial nerve VIII.

In the one patient with myelitis, 6 contrast enhancing lesions were detected in the cervical and thoracic spinal cord. This patient suffered from weakness and numbness/paresthesia in the left arm and numbness/paresthesia below level Th10.

#### Postherpetic neuralgia

Sixteen patients (6%) were hospitalized due to postherpetic neuralgia as consequence of trigeminal or dorsal root ganglionitis. Neuralgia was mostly found in the thoracic region (6 patients, 38%) and trigeminal region (5 patients, 31%). Three patients (19%) presented with cervical neuralgia and 2 patients (13%) with lumbar neuralgia. The gender distribution was 56% females to 44% males. In all but one patient, neuralgia occurred in older patients (> 60 years).

### Comorbidities in patients with VZV infection

**Fig. 3:**
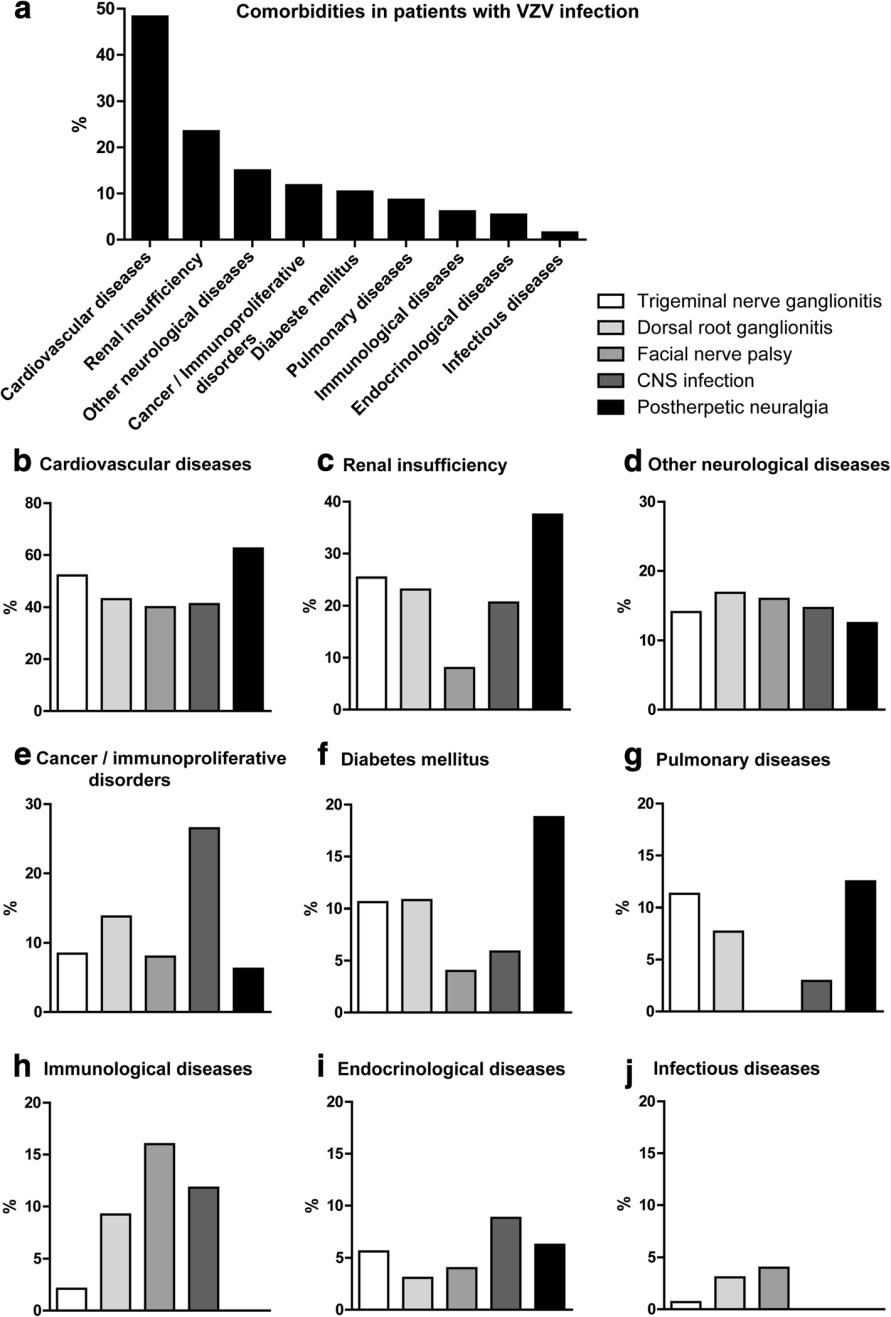
Distribution of comorbidities in patients with varicella zoster virus reactivation. **a** shows the distribution of all comorbidities in patients with varicella zoster virus reactivation. **b**-**j** illustrate the distribution of comorbidities separately in patients with varicella zoster virus reactivation. Graphs show the percentage frequency of comorbidities

The distribution of comorbidities in patients with a VZV disease is shown in Fig. 3. In patients with rash the presence of cancer or immunoproliferative disorders was associated with an increased probability for a CNS infection as compared to all patients with rash (OR 3.123, CI 1.14–7.97, *p* = 0.0338). In contrast, patients with rash and concomitant immunological diseases did not differ significantly from all patients with rash concerning the occurrence of CNS infection (*p* = 0.18).

Patients with additional cardiovascular diseases suffered from arterial hypertension (88%), coronary artery disease (22%), arrhythmia (15%), and/or chronic heart failure (6%). Patients with additional other non-inflammatory neurological diseases suffered from encephalopathy/dementia (38%), epilepsy (28%), polyneuropathy (25%), Parkinson’s disease (5%), residual symptoms after stroke (5%), and motorneuron disease (3%). Cancer or immunoproliferative disorders included 15 solid tumors (47%) and 17 immunoproliferative disorders (53%). Patients with additional lung diseases suffered from chronic obstructive lung disease (54%), asthma (33%), emphysema (8%), or fibrosis (4%). Patients with additional endocrinological diseases suffered from hypothyreosis (80%), hyperthyreosis (13%), or hyperparathyroidism (7%). Patients with additional immunological diseases suffered from rheumatoid arthritis (6 patients, 35%), lung sarcoidosis (2 patients), giant cell arteritis (1 patient), Hashimoto’s thyreoiditis (1 patient), multiple sclerosis (1 patient), myasthenia gravis (1 patient), cerebral vasculitis (1 patient), temporal arteritis (1 patient), Crohn’s disease (1 patient), ulcerative colitis (1 patient). Four patients with infectious diseases were identified: hepatitis B in 2 patients, HIV in 1 patient, and syphilis in 1 patient.

### Lymphocyte counts in patients with immunoproliferative and immunological diseases

Patients with immunoproliferative diseases showed lymphocyte counts with a median of 0.9 × 10^9^/L (range 0.4–19.2 × 10^9^/L). In 12/17 patients blood lymphopenia (< 1.5 × 10^9^/L) was detected with a median of 0.8 × 10^9^/L (range 0.5–1.4). One other patient exhibited 19.2 × 10^9^/L lymphocytes which led to the diagnosis of lymphatic leukemia. Eight of 17 patients with immunoproliferative diseases were treated with immunosuppressive therapies (prednisolone, rituximab, cyclophosphamide, bendamustine, adriamycin, vincristine, fludarabine) during the time of VZV infection. Three other patients were diagnosed with immunoproliferative disorders during hospitalization due to VZV infection.

Patients with autoimmune diseases exhibited lymphocyte counts with a median of 1.5 × 10^9^/L (range 0.3–2.8 × 10^9^/L). 8/17 patients showed blood lymphopenia (< 1.5 × 10^9^/L) with a median of 0.7 × 10^9^/L (range 0.3–1.2). Ten of 17 patients with autoimmune diseases (6 patients with rheumatoid arthritis, 1 patient with giant cell arteritis, 1 patient with Crohn’s disease, 1 patient with myasthenia gravis, 1 patient with cerebral vasculitis) were treated with immunosuppressive therapies (prednisolone, azathioprine, methotrexate) during the time of VZV infection.

### Serological IgG VZV-values in patients with VZV infection

VZV-IgG measurements were performed in 11/142 patients with trigeminal nerve ganglionitis, in 15/65 patients with dorsal root ganglionitis, in 18/25 patients with facial nerve palsy, and in 27/34 patients with CNS infection. Patients with trigeminal nerve ganglionitis exhibited VZV-IgG values with a median of 1650 IU/l (range 190–8500 IU/l). In patients with facial nerve palsy median values of 1937 IU/l (range 190–7193 IU/l) were found. Patients with dorsal ganglionitis exhibited median values of 3258 IU/l (range 350–5400 IU/l), while patients with CNS infection displayed median values of 5481 IU/l (range 880–9600).

### General signs of infection in patients with VZV disease

At admission, fever (> 38 °C) was found more frequently in patients with CNS infection (Table 1).

In patients with rash the presence of fever was associated with an increased probability for a CNS infection as compared to all patients with rash (OR 7.921, CI 2.71–23.19, *p* = 0.005). Still, elevated body temperature was found in only 29% of patients with CNS infection. In contrast, patients with rash and elevated CRP (C-reactive protein) values did not differ significantly from all patients with rash concerning the occurrence of CNS infection (*p* = 0.089).

### Brain imaging in patients with CNS infection

Brain MRI was performed in 15/18 patients with encephalitis (12 with contrast enhancement) and 10/15 patients with meningitis (all ten with contrast enhancement). One patient with encephalitis who presented with clinical affection of multiple cranial nerves (VII, IX, X, and XII) showed contrast enhancement in the hypoglossal nerve. All other 24 patients did not show any signs of infection in the brain parenchyma. CT of the brain was performed in the other 3 patients with encephalitis and 3 patients with meningitis and did not reveal any pathological signs of inflammation. Only two patients with meningitis did not receive brain imaging. MRI showed contrast enhancement in the one patient with myelitis.

### Complications during disease course in patients with CNS infection

Eight of 18 patients with encephalitis developed severe complications. One patient died on day eight of antiviral treatment, while another 7 patients needed rehabilitation. Six of them showed severe neuropsychiatric symptoms and one developed pericarditis.

After 7 to 53 days of antiviral treatment, another 5 patients (2 with encephalitis and 3 with meningitis) developed diplopia due to oculomotor disturbances. Three patients presented with abducens nerve palsy. One of them showed a new brainstem lesion in the pons, while in the 2 other patients MRI did not reveal any changes as compared to MRI at presentation. One patient showed a complex eye movement disturbance due to intra- and periorbital oedema as seen on MRI. Another patient presented with oculomotor nerve palsy without any MRI changes. Considering autoimmune processes after viral infection, patients were treated with steroids, which led to improvement of symptoms.

### Cerebrospinal fluid findings

#### Trigeminal nerve ganglionitis

**Fig. 4:**
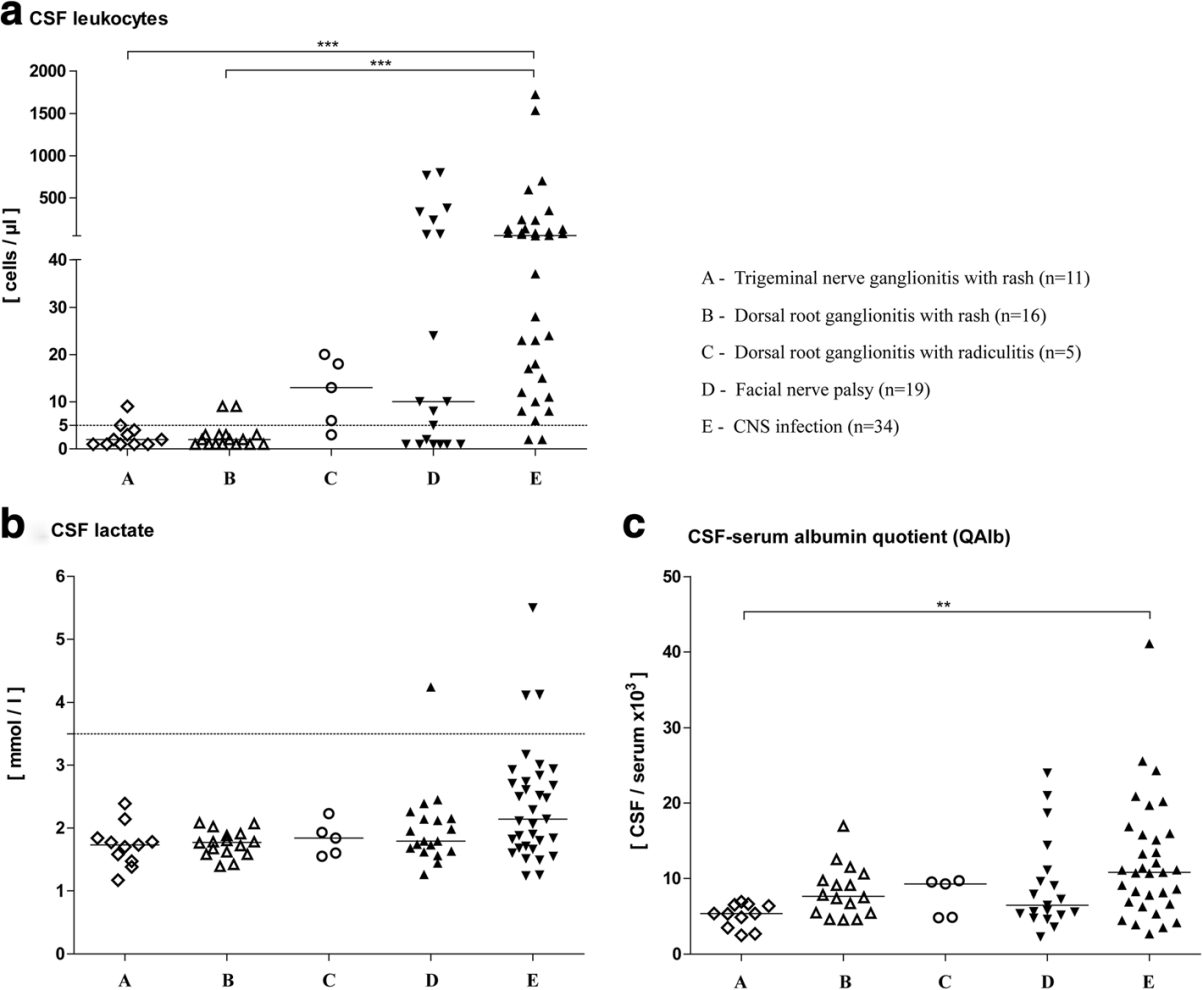
Cerebrospinal fluid results in patients with varicella zoster virus reactivation. Graphs show the distribution of cell count (**a**), lactate (**b**), and albumin CSF/serum quotients (**c**). Bars represent median values in each group. Cell count ≥5/μl and lactate ≥3.5 mmol/l were considered elevated. **P < 0.01, ***P < 0.001

Among the patients with trigeminal nerve ganglionitis only marginal CSF changes were found. Two patients (18%) were identified with a slightly increased leukocyte cell count of 5 and 9 cells/μl, respectively (Table 2, Fig. 4). In all patients, normal blood–CSF barrier function and lactate concentrations were found. There was no quantitative intrathecal synthesis of immunoglobulins. Only one patient exhibited CSF oligoclocal bands (9%). VZV virus was neither directly (PCR) nor indirectly (VZV specific intrathecal antibody) detected in the CSF.

#### Dorsal root ganglionitis

In patients with dorsal root ganglionitis with isolated segmental rash some CSF changes were found as 2 patients exhibited a slight pleocytosis of 9 cells/μl (13%, Table 2, Fig. 4). A blood–CSF barrier dysfunction was detected in 8 patients (50%). In one patient with elevated cell count, VZV DNA was identified in the CSF, while another exhibited elevated VZV specific intrathecal antibody synthesis. Intrathecal synthesis of immunoglobulins did not occur.

In 4/5 patients with dorsal root ganglionitis and clinical signs of nerve affection due to radiculitis a marginally elevated cell count of 6–20 cells/μl (80%) was detected. QAlb values were slightly increased in 3 of them (60%). Quantitative intrathecal synthesis of immunoglobulins was not found. Only 1 patient showed CSF oligoclonal bands. In 3 patients with pleocytosis, a VZV infection was detected (1 patient had a positive VZV DNA and 2 showed virus specific intrathecal antibody synthesis). Lactate concentrations were normal in all patients.

#### Facial nerve palsy

Twelve patients (63%) were identified with increased cell counts ranging from 5 to 800 cells/μl (Table 2, Fig. 4). A blood–CSF barrier dysfunction was found in 8 patients (42%). CSF lactate was elevated in 1 patient (5%). Quantitative intrathecal synthesis of immunoglobulins was found in 4 patients (21%). Seven patients exhibited oligoclocal bands restricted to the CSF (37%). VZV specific CSF changes were found in 12 patients (63%). Three patients were identified by VZV DNA and 9 by VZV specific intrathecal antibody synthesis. Clinical signs of CNS infection were not found in these patients.

#### CNS infection (encephalitis, meningitis, myelitis)

In all but 2 patients, increased cell counts ranging from 6 to 1720 cells/μl were found (94%, Table 2, Fig. 4). Two patients with normal cell counts had an underlying malignancy of whom one received chemotherapy 3 weeks before. A blood–CSF barrier dysfunction was found in 20 patients (59%). Only 3 patients presented increased CSF lactate concentrations (9%). Quantitative intrathecal synthesis of immunoglobulins was found in 6 patients (18%). Eleven patients (32%) showed oligoclonal bands restricted to the CSF. CSF infection with VZV was verified in all patients (100%). Twenty-three patients were identified by positive PCR results and 11 patients by VZV specific intrathecal antibody synthesis. All of these patients were additionally examined and found negative for enteroviruses, herpes simplex virus, cytomegalovirus, Epstein-Barr virus.

Statistics revealed that pleocytosis (*p* = 0.001) and VZV-positive findings in the CSF upon virological diagnostics (*p* = 0.0001) were most frequently found in patients with involvement of the CNS as compared the other groups.

## Discussion

Here we illustrate characteristics of clinical and immunological features in patients with VZV reactivation. The age distribution in our cohort with sharply rising incidence after the age of 50 and peaking in the eighth decade of life confirms that herpes zoster is primarily a disease of the elderly [5]. VZV reactivation typically manifests as a rash and ganglionitis. In our in-patient cohort trigeminal nerve involvement affecting the ophthalmic branch was the most common clinical manifestation followed by thoracic and cervical ganglionitis. This observation is in contrast to previous reports as it has been reported inversely [27, 28]. A possible explanation might be that truncal zoster is mostly treated in an outpatient setting.

Although monosegmental herpes zoster is often uncomplicated, several complications of the central and peripheral nervous system may occur and some of them may not be accompanied by rash. In our cohort, rash was absent in a quarter of patients with CNS involvement and facial neve palsy. Our results are similar to a previous study showing that rash was absent in 15% of patients with definite VZV mediated facial nerve palsy [29]. Since antiviral treatment concerning dosage and duration vary between patients with uncomplicated herpes zoster and patients with cranial nerve or CNS involvement a fast diagnosis is required. In our cohort, patients with CNS involvement frequently developed severe complications, and thus, sufficient therapy is crucial. However, brain imaging failed to detect CNS infection and general signs of infection such as fever or elevated CRP values were absent in nearly every second patient. This underlines the importance of early CSF analysis. Although slight inflammatory CSF changes can be found in patients with isolated herpes zoster because of the close anatomical relation of ganglions and the CNS, the frequency is clearly higher in patients with CNS involvement. In our cohort, pleocytosis was found in only very few patients with isolated/segmental herpes zoster with cell numbers lower than 10/μl. In contrast, higher pleocytosis frequencies were found in patients with neuronal affection due to radiculitis or facial nerve palsy and in patients with CNS involvement. Similar to pleocytosis virus detection in the CSF was rare in patients with isolated herpes zoster but occurred frequently in patients with cranial nerve or CNS affection. All patients with CNS involvement were tested positive for VZV in CSF. PCR is a very sensitive method for detecting of virus genomes in the CSF. However, 7–10 days after infection, production of virus specific antibodies in the CSF becomes detectable, whereas PCR analysis might already reveal negative results at that time [30]. In our cohort, the majority of patients with CNS infection were identified by positive PCR results (23/34) while eleven patients were identified by VZV specific intrathecal antibody synthesis due to different disease duration. While detection of VZV in CNS infection by PCR yielded positive results in two thirds of the patients, it was less effective in facial nerve palsy. Previous recommendations to rely on PCR only and to exclude investigations in patients with CSF cell counts < 10/μl [31] are thus not justified.

It is controversially discussed if VZV directly induces real encephalitis with viral damage of neurons or rather an encephalopathy due to vasculopathy. VZV is known to replicate in arteries resulting in vasculopathy. In patients deceased due to VZV encephalitis pathological examinations of the brain revealed signs of inflammatory changes around vessels predominantly in the grey matter. In a pediatric case swellings of capillary and venular endothelium and inflammatory cellular reactions including lymphocytes, plasma cells, and microglia around larger vessels were found [32]. In our cohort, all patients with encephalitis but one exhibited disturbance of consciousness and/or neuropsychiatric symptoms. The majority did not exhibit focal neurological symptoms, which is more suggestive of a diffuse encephalopathy rather than focal encephalitis. In agreement with this brain imaging did not show any signs of infection in all but one patient with encephalitis. The imaging findings are in line with the observation of others [12] and are in contrast to abnormalities frequently found in patients with herpes simplex encephalitis [33].

Our study has some limitations. Data were evaluated retrospectively, and thus, CSF examinations are not available for all patients. Data are available for patients who were hospitalized, and thus, the distribution of herpes zoster with predominant affection of the trigeminal ganglion might be overestimated compared to an outpatient setting. Furthermore, evidence from the last decade suggests that VZV induced vasculopathy may be a cause of stroke and cerebral thrombosis [27, 34] and infection of the temporal arteries may mimic giant cell arteritis [35]. Our retrospective study population did not contain such patients because investigation of the CSF was estimated to be unnecessary in this population group.

## Conclusions

Our results show that CSF analysis is essential as a diagnostic tool in patients with VZV infection and neuro-psychiatric symptoms. Skin lesions were lacking in a quarter of patients with CNS involvement and facial nerve palsy in our cohort. Therefore, physicians should be aware, that in these cases VZV infection can only be proven by CSF analysis and -due to frequent and severe complications in encephalitis- a quick diagnosis is crucial.

## Abbreviations

CNS: Central nervous system
CSF: Cerebrospinal fluid
MRI: Magnetic resonance imaging
OCB: CSF-specific oligoclonal bands
QAlb: CSF-serum albumin quotients
VZV: Varicella zoster virus
